# A case report of myocarditis secondary to eosinophilic granulomatosis with polyangiitis

**DOI:** 10.1093/ehjcr/ytac307

**Published:** 2022-07-25

**Authors:** Dorina-Gabriela Condurache, Zahra Raisi-Estabragh, Rohit Baslas, Shahir Hamdulay

**Affiliations:** London North West University Healthcare NHS Trust, Watford Road, Harrow HA1 3UJ, UK; William Harvey Research Institute, NIHR Barts Biomedical Research Centre, Queen Mary University London, Charterhouse Square, London EC1M 6BQ, UK; Barts Heart Centre, St Bartholomew’s Hospital, Barts Health NHS Trust, West Smithfield, EC1A 7BE, London, UK; London North West University Healthcare NHS Trust, Watford Road, Harrow HA1 3UJ, UK; London North West University Healthcare NHS Trust, Watford Road, Harrow HA1 3UJ, UK

**Keywords:** Eosinophilic granulomatosis with polyangiitis, Vasculitis, Acute myocarditis, Case report

## Abstract

**Background:**

Eosinophilic granulomatosis with polyangiitis (EGPA) is a rare form of anti-neutrophil cytoplasm antibody (ANCA)-associated vasculitis. Cardiac involvement is the major cause of morbidity and mortality in these patients. Early recognition and treatment initiation for such manifestations are key to improved patient outcomes.

**Case summary:**

We report the case of a 60-year-old man with a history of therapy-resistant asthma and rhinitis. He presented with acute chest pain, sinus tachycardia, and marked peripheral eosinophilia. Transthoracic echocardiogram (TTE) showed segmental anterior left ventricular (LV) wall motion abnormalities with impaired systolic function (LV ejection fraction 45%) and a small pericardial effusion. Invasive coronary angiography revealed unobstructed coronary arteries. Cardiac magnetic resonance imaging confirmed the TTE findings and demonstrated oedema and active inflammation of the anterior and anteroseptal LV segments [Short inversion time recovery (STIR)-T2] and an unusual pattern of non-ischaemic late gadolinium enhancement extending across multiple coronary territories. Autoantibody testing detected a positive *P*-ANCA and myeloperoxidase (MPO) antibodies. Overall, the investigation findings supported a diagnosis of ANCA-positive EGPA with acute myocardial involvement. He was initially treated with high-dose corticosteroids, cyclophosphamide, and rituximab. The patient had a good symptomatic and biochemical (normalized troponin T and MPO titre) recovery. In addition, subsequent TTE showed improvement of LV systolic function and resolution of regional wall motion abnormalities.

**Discussion:**

In this case, prompt diagnosis facilitated early initiation of immunosuppressive therapy and disease remission. CMR provides non-invasive assessment of myocardial tissue characterization and, used in conjunction with other tools, can be instrumental in detecting myocardial involvement in EGPA.

**Learning points**

- Eosinophilic granulomatosis with polyangiitis (EGPA) is an anti-neutrophil cytoplasm antibody (ANCA)-associated vasculitis with cardiac involvement as the major cause of mortality and morbidity.
- Red flags include unexplained dyspnoea, palpitations, chest pain with or without increased troponin, syncope, arrhythmia and acute or chronic congestive heart failure, aborted sudden cardiac death and fulminant cardiogenic shock.
- Cardiovascular magnetic resonance (CMR) imaging provides a non-invasive assessment of myocardial characteristics and is a key tool in the diagnosis of acute myocarditis. Interpreted in conjunction with other tests it can be instrumental in diagnosis of myocardial involvement in EGPA.
- Contemporary medical therapy, including corticosteroids, cyclophosphamide, and rituximab can induce complete remission.

## Introduction

Eosinophilic granulomatosis with polyangiitis (EGPA), also known as Churg–Strauss syndrome, is a systemic vasculitis characterized by eosinophil-rich granulomatous inflammation of small to medium-sized arteries which may be associated with anti-neutrophil cytoplasm antibody (ANCA) antibodies. EGPA is one of the most common systemic vasculitides which can affect the heart. The reported frequency of cardiac involvement in these patients varies between 16.0 and 29.0% in different studies^1,2,3^ and is a major cause of morbidity and mortality. The condition is infrequently encountered, and detection of cardiac involvement poses diagnostic challenges. Early recognition and initiation of treatment can positively alter the disease trajectory, preserve cardiac function and reduce associated mortality. We report the case of a 60-year-old man diagnosed with EGPA associated myocarditis—highlighting the diagnostic, treatment, and follow-up in the clinical pathway.

## Timeline

**Table:** 

Timing	Key events
Chronic history	Allergic rhinitis
3 years prior	New diagnosis of therapy-resistant asthma (age 57 years)
Day 0	Acute chest pain, non-specific T wave changes, significantly elevated Troponin T (1263 ng/L). No culprit lesion on invasive coronary angiogram, no lung pathology on chest x-ray. Very high eosinophil count of 27.3% 5.0 × 10^9^/L.
Day 0	Echocardiogram revealed segmental anterior left ventricular (LV) wall motion abnormalities, mildly reduced LV systolic function, and small pericardial effusion. NT-pro BNP was elevated at 6470 ng/L.
Day 1	Cardiac magnetic resonance (CMR) confirmed echocardiographic findings of LV wall motion abnormalities and systolic impaired, and additionally demonstrated active oedema and inflammation in the anterior and anteroseptal segments suggestive of myocarditis. Patient commenced on high-dose oral prednisolone for working diagnosis of vasculitic myocarditis.
Day 4	Electromyography showed no evidence of myositis.
	Myeloperoxidase antibodies level was 22 IU/mL (normal range: 0–5 IU/mL).
Day 6	Cyclophosphamide initiated due to steroids-induced psychosis. Prednisolone dose decreased.
Day 12	Rituximab administered with plan for commencing a twice weekly regimen.
Day 14	Repeat Echocardiogram showed improvement of LV systolic function.
Day 20	Second dose of cyclophosphamide administered.
Day 34	Improvement in symptoms, blood biomarker, and imaging findings. Thus, discharged with plan for close outpatient follow-up. Plan for reducing dose of prednisolone and stopping over 12-weeks.
Day 65	Third dose cyclophosphamide administered.
Day 100	Fourth dose of cyclophosphamide given.
Day 120	Patient in remission on oral azathioprine and shows no evidence of ongoing myocarditis.

## Case summary

**Figure 1:**
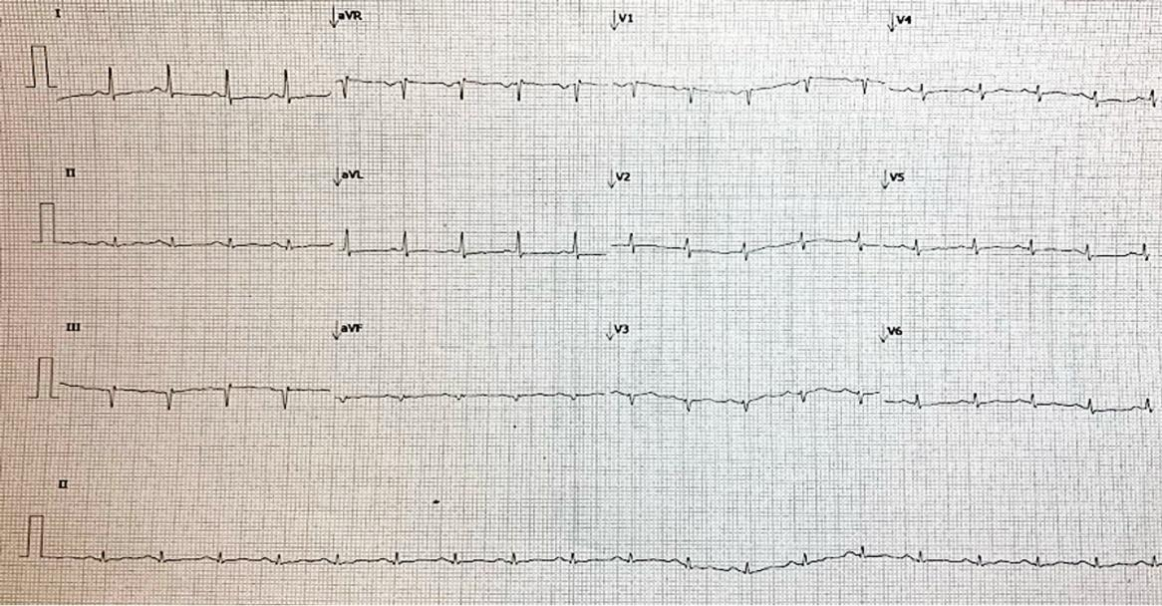
Twelve-lead resting electrocardiogram on admission demonstrating sinus tachycardia and global *t* wave flattening.

A 60-year-old man with a past medical history of therapy-resistant asthma and rhinitis presented to the emergency department with a history of severe chest pain, nausea, and sweating. He also gave a history of neck pain, fatigue, and generalized weakness. He had no history of cardiac disease or conventional cardiovascular risk factors. Physical examination showed a blood pressure of 100/60 mmHg with a heart rate of 130 beats/min, normal heart sounds and a clear chest. A 12-lead resting electrocardiogram showed sinus tachycardia with globally flat T-waves (*Figure 1*); there were no dynamic ST segment changes. Blood tests revealed leucocytosis with elevated WCC of 18.16 × 10^9^/L (normal value 3–10.0 × 10^9^/L) and marked peripheral eosinophilia of 27.3% 5.0 (normal range between 0.0–0.4^10^9^/L). Inflammatory markers were raised with C-reactive protein (CRP) at 92 mg/L (normal value: 0.05–5.0 mg/L) and Erythrocyte sedimentation rate (ESR) of 22 mm/h (normal value: 1–20 mm/h). Troponin T was raised at 1267 ng/L (normal range 0–14 ng/L) and *N*-terminal pro B-type natriuretic peptide (NT-pro BNP) raised at 6470 ng/L (normal value: 133–450 ng/L) (*Table 1*).

**Table 1:** Summary of blood biomarker trends from admission to first outpatient follow-up

	Reference range	Day 0 admission	Day 14 (in-hospital)	Day 34 (discharge)	Day 120 (follow-up)
WBC (× 10^9^/L)	3.0–10.9	18.4	11.3	9.4	6.1
Eosinophil count (× 10^9^/L)	0.0–0.4	27.3% 5.0	11.7% 1.4	8.1% 0.8	3.8% 0.2
Hb (g/L)	130–170	129	147	137	149
Troponin (ng/L)	0–14	1263	130	40	10
NT-pro BNP (ng/L)	133–450	6470	1350	747	152
ESR (mm/hour)	1–20	22	11	13	2
CRP (mg/L)	0.0–5.0	92	58	1.9	5.2
MPO Titre (IU/mL)	0–5	22	—	0.9	0.5

ESR = erythrocyte sedimentation rate, CRP = C-reactive protein, Hb = haemoglobin, MPO = myeloperoxidase antibodies titre, NT-pro BNP = B-type natriuretic peptide, WBC = white blood cells

**Figure 2:**
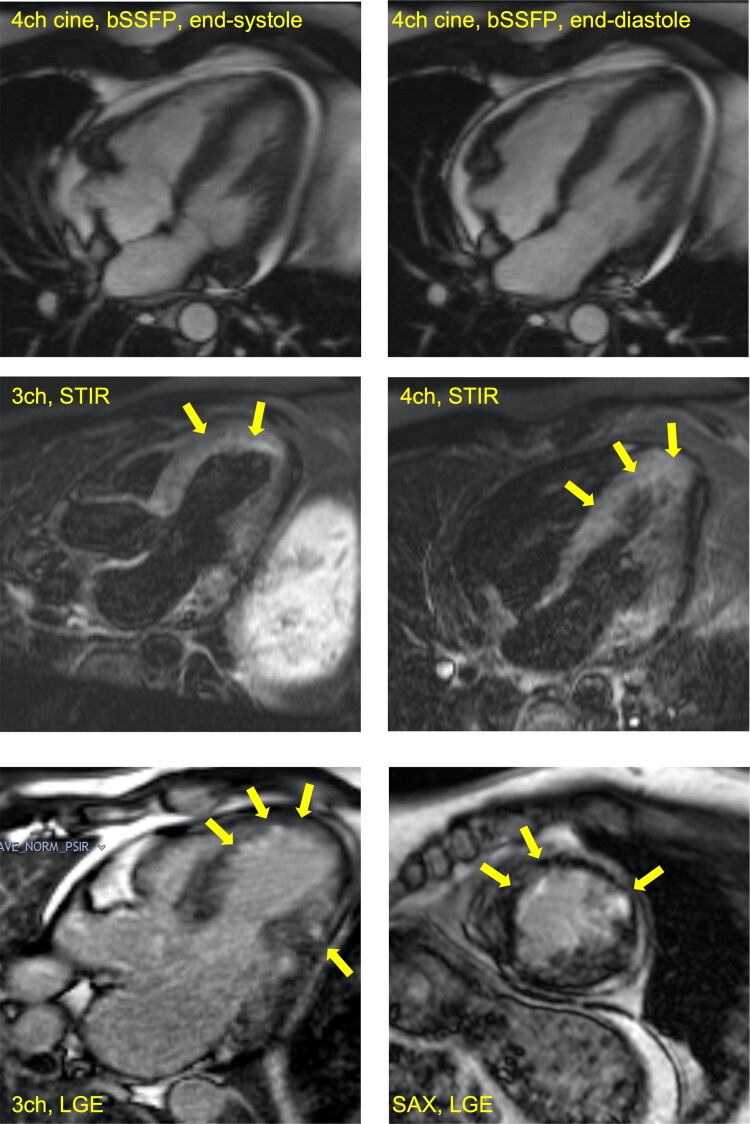
Key cardiovascular magnetic resonance images demonstrating myocardial oedema and subendocardial LGE without coronary distribution footnote. 3ch, three chamber; 4ch, four chamber; bSSFP, balanced steady state free precession; LGE, late gadolinium enhancement; SAX, short axis; STIR, short inversion time inversion recovery; Panels A and B. Four-chamber long-axis view frames at end-diastole (*A*) and end-systole (*B*) demonstrating reduced left ventricular function. Panel C. T2w-STIR-3 chamber showing enhancement in the anterior and anteroseptal segments. Panels C to D. EGE (early gadolinium enhancement) and LGE (late gadolinium enhancement) images demonstrating subendocardial contrast uptake in a non-ischaemic distribution.

Transthoracic echocardiography (TTE) revealed segmental anterior left ventricular (LV) wall motion abnormalities with impaired systolic function (LV ejection fraction 45%) and a small pericardial effusion measuring 0.64 cm anteriorly and 1.1 cm to the rear of the right atrium without haemodynamic compromise. Invasive coronary angiography showed unobstructed coronary arteries. Cardiovascular magnetic resonance (CMR) demonstrated borderline hypertrophy of the LV septal wall (thickness12–12.5 mm), regional hypokinesia of the anterior and anteroseptal LV segments extending from base to apex, mildly impaired LV systolic function (LV ejection fraction 48%), and a small pericardial effusion (*Figure 2*). T2w-STIR CMR images demonstrated significant enhancement in the anterior and anteroseptal segments, indicating myocardial oedema and active inflammation in these regions (*Figure 2*). Late gadolinium enhancement demonstrated subendocardial contrast uptake in a non-ischaemic distribution (*Figure 2*). Specifically, there was subendocardial enhancement in the mid-apical anteroseptum and anterior walls, the basal to mid lateral wall, and focal near transmural enhancement in the mid anterolateral wall.

Overall, the investigation findings supported a non-ischaemic pattern of active myocardial inflammation. In the context of the very high eosinophil count, a vasculitic process was considered as the working diagnosis. A computed tomography (CT) scan of the neck, thorax, abdomen, and pelvis was unremarkable; in particular showing no significant lymphadenopathy or pulmonary abnormalities. A 24 h Holter monitor excluded any ventricular arrythmias.

Further work up for vasculitis resulted in detection of positive *P*-ANCA and positive myeloperoxidase (MPO) antibodies. The clinical findings were consistent with a diagnosis of EGPA and myocarditis. The patient was started on oral prednisolone 60 mg OD. Although he responded well to steroids, he developed steroid-induced psychosis. In view of this, prednisolone dose was decreased to 30 mg OD and he was commenced on intravenous cyclophosphamide therapy (1000 mg each dose) and Rituximab (1 g, 2 weeks apart). This was followed by a rapid improvement in symptoms (within 2 weeks), with improvement in inflammatory markers, eosinophil count, NT-pro BNP and troponin T (*Table 1*). Subsequent TTE showed improvement of his LV systolic function and no regional wall motion abnormalities. He received a further four cycles of cyclophosphamide (six doses of 1000 mg each) with tapering oral prednisolone and was initiated on azathioprine as a maintenance agent. After 2 years of follow-up, the patient remains in remission with normal BNP, Troponin T, negative ANCA, and normal eosinophil counts.

## Discussion

EGPA (previously known as Churg–Strauss syndrome) is a rare, systemic, necrotizing small-vessel vasculitis with accompanying bronchial asthma, eosinophilia and eosinophilic tissue infiltration of various tissues with granuloma formation.^4^ Clinical presentation of myocarditis is non-specific. Red flags may include unexplained dyspnoea, palpitations, chest pain with or without increased troponin, syncope, arrhythmia and acute or chronic congestive heart failure, aborted sudden cardiac death, and fulminant cardiogenic shock.^1^ The differential diagnosis for eosinophilic myocarditis includes hypersensitivity myocarditis, EGPA, and Loeffler endomyocarditis (hypereosinophilic syndrome). The distinction among these entities is generally made based on clinical grounds.^5^ EGPA is one of the most common systemic vasculitides which affect the heart.^6^ and if present is associated with high mortality and a poor prognosis. When EGPA affects the heart, it can lead to a myriad of presentations such as myocarditis with cardiomyopathy, pericarditis with pericardial effusion (up to 25% of patients), heart failure (18%), various ventricular and supraventricular arrhythmias, valve involvement,^7^ and sudden cardiac death.^8,9^

Vasculitis related ischaemia and eosinophilic infiltration of the myocardium^8^ are the two main mechanisms involved in cardiac EGPA. Epicardial coronary artery involvement is rare;^9^ however, coronary angiography should be considered in patients presenting with angina symptoms to rule out coronary vessel stenosis.

Imaging techniques such as TTE and CMR may help in establishing the diagnosis of EGPA. TTE is an excellent first line modality, allowing rapid assessment of ventricular function. CMR is the reference standard for volumetric chamber quantification, providing more reproducible assessments of ventricular function and permits non-invasive myocardial tissue characterization, including the presence of myocardial oedema, inflammation, and fibrosis.^10^ Cardiovascular magnetic resonance has emerged as a sensitive and non-invasive technique in the evaluation of cardiac lesions in EGPA patients. Garcia-Vives *et al*.^11^ demonstrated in their study that 45% of asymptomatic patients had an abnormal baseline cardiac evaluation. Similarly, Pakbaz *et al*.^12^ studied 62 cases in active disease and demonstrated that cardiac symptoms, electrocardiographic abnormalities, abnormal biomarkers, and abnormal echocardiography were detected in 82.3, 68.5, 77.4, and 96.8%, respectively. In patients with active EGPA, CMR enables detection of cardiac involvement even when cardiac symptoms are not present,^13^ and these studies recommend prompt cardiac screening in all EGPA patients, instead of a symptoms-guided algorithm.

Our patient fulfilled the recent 2022 ACR/EULAR Classification Criteria for EGPA.^14^ The pathognomonic laboratory feature of EGPA is prominent peripheral eosinophilia that commonly exceeds 1500 cells/μL. ANCA is present in ∼40% of patients showing a perinuclear staining pattern (*P*-ANCA) mostly directed against myeloperoxidase (anti-MPO).^7^ In the present case, CMR had a key role in establishing the correct diagnosis, providing complementary and additive information to the clinical picture. The CMR findings (regional wall motion abnormalities, impaired LV function, positive STIR, subendocardial LGE without coronary distribution) in the context of late onset asthma and rhinitis, peripheral eosinophilia, elevated troponins and inflammatory markers (ESR, CRP), with unobstructed coronaries was consistent with the diagnosis of myocardial EGPA.

International recommended treatment for EGPA are systemic glucocorticoids (prednisone) at a dose of 0.5 to 1 mg/kg per day, cyclophosphamide or rituximab as induction agents, and azathioprine or methotrexate as maintenance agents.^15^ For patients with severe vasculitis (impending respiratory failure, cardiac involvement, glomerulonephritis, and neuropathy) and acute multi-organ disease, IV glucocorticoids (methylprednisolone) at a dose of 1 g daily for 3 days is given followed by oral glucocorticoid therapy.^14,15,16^ Our patient was started on oral prednisolone 60 mg OD. Although he responded well to steroids, he developed steroid-induced psychosis. In view of this, prednisolone dose was decreased to 30 mg OD, and he was commenced on intravenous cyclophosphamide therapy (1000 mg each dose) and rituximab (1 g, 2 weeks apart).

In this case, prompt diagnosis with the aid of CMR facilitated treatment with prednisolone, rituximab, and cyclophosphamide and prompt remission of EGPA and associated myocarditis. Cardiovascular magnetic resonance is a safe non-invasive assessment that can be applied across the spectrum of myocarditis irrespective of aetiology. Cardiovascular magnetic resonance data considered in conjunction with clinical and laboratory findings can reliably support a unifying diagnosis without performing an invasive endomyocardial biopsy. While the diagnosis of vasculitis can be established on clinical grounds supported by characteristic radiographic and serological testing, tissue acquision is sometimes necessary when the diagnosis is uncertain. Endomyocardial biopsies are occasionally done, but given the potential risks and low diagnostic yield, it is used sparingly. It may be particularly useful for diagnosis if investigations suggest non-ischaemic myocardial involvement, if there is unexplained or an unexpected change in cardiac status, and if histological confirmation is expected to change management.

## Conclusions

We present a rare case of eosinophilic myocarditis in a patient with EGPA. Cardiovascular magnetic resonance played a crucial role in the diagnosis of this rare presentation. Early detection and treatment of cardiac involvement in EGPA are key to achieving complete recovery and minimising long term sequalae.

## Supplementary Material

ytac307_Supplementary_Data

## Lead author biography

Dorina-Gabriela Condurache graduated from Grigore T. Popa University of Medicine and Pharmacy, Iași, Romania. Currently, she is a junior registrar with a keen interest in cardiology, academic research and medical education.

**Slide sets:** A fully edited slide set detailing this case and suitable for local presentation is available online as Supplementary data.

**Consent:** Consent for publication has been obtained, in line with the COPE best practice guidelines, and that the individual who is being reported on is aware of the possible consequences of that reporting.

## Notes

**Funding:** Z.R.-E. recognizes the National Institute for Health Research (NIHR) Integrated Academic Training programme which supports her Academic Clinical Lectureship post.
