# Supplementary material for: A case report of myocarditis secondary to eosinophilic granulomatosis with polyangiitis
Source: Eur Heart J Case Rep. 2022 Jul 25;6(8):ytac307. doi: 10.1093/ehjcr/ytac307 (PMC9426485; doi:10.1093/ehjcr/ytac307)
Supplement: ytac307_Supplementary_Data [file ytac307_supplementary_data.pptx]

## Slide 1
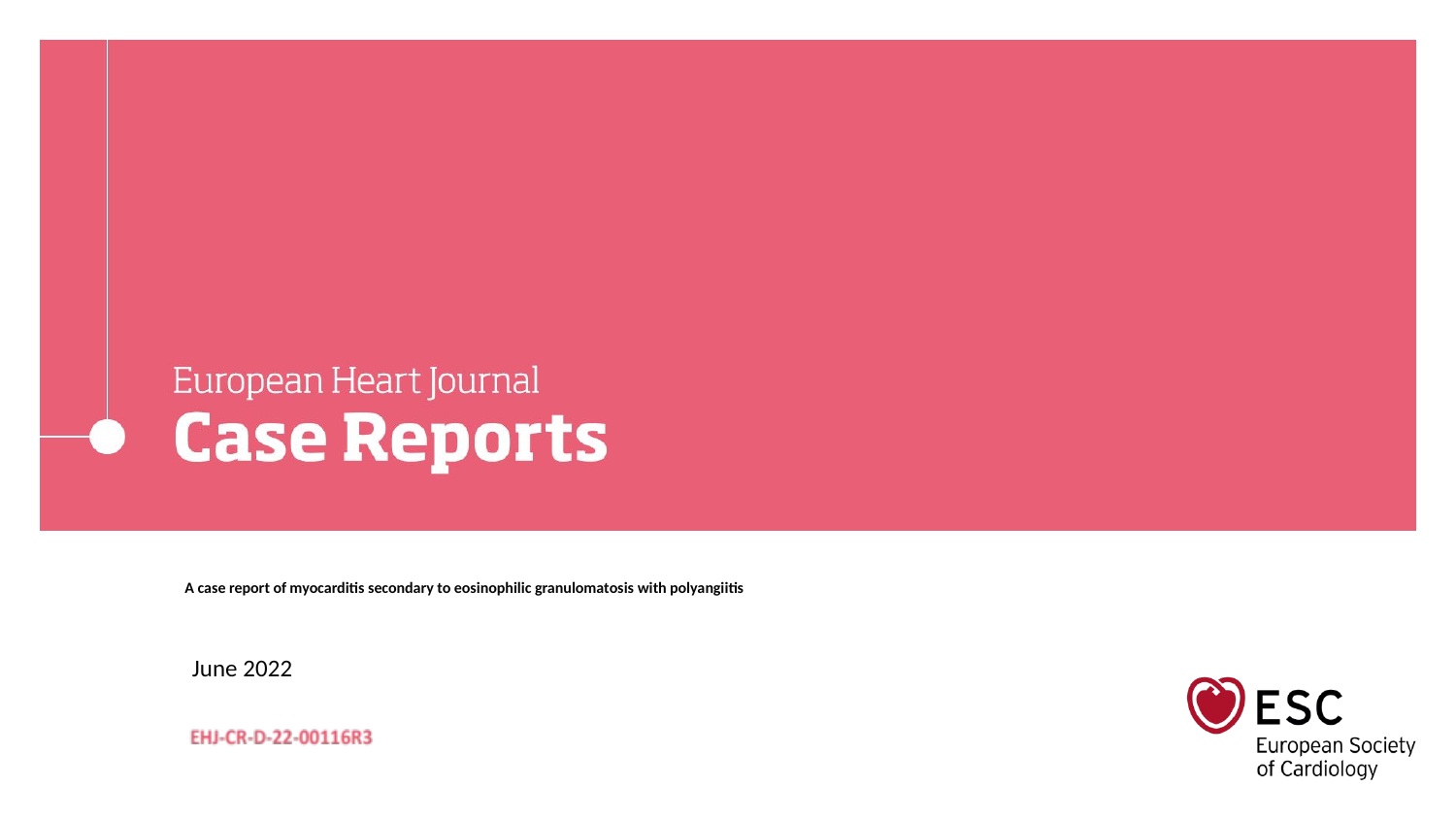

# A case report of myocarditis secondary to eosinophilic granulomatosis with polyangiitis
June 2022

## Slide 2
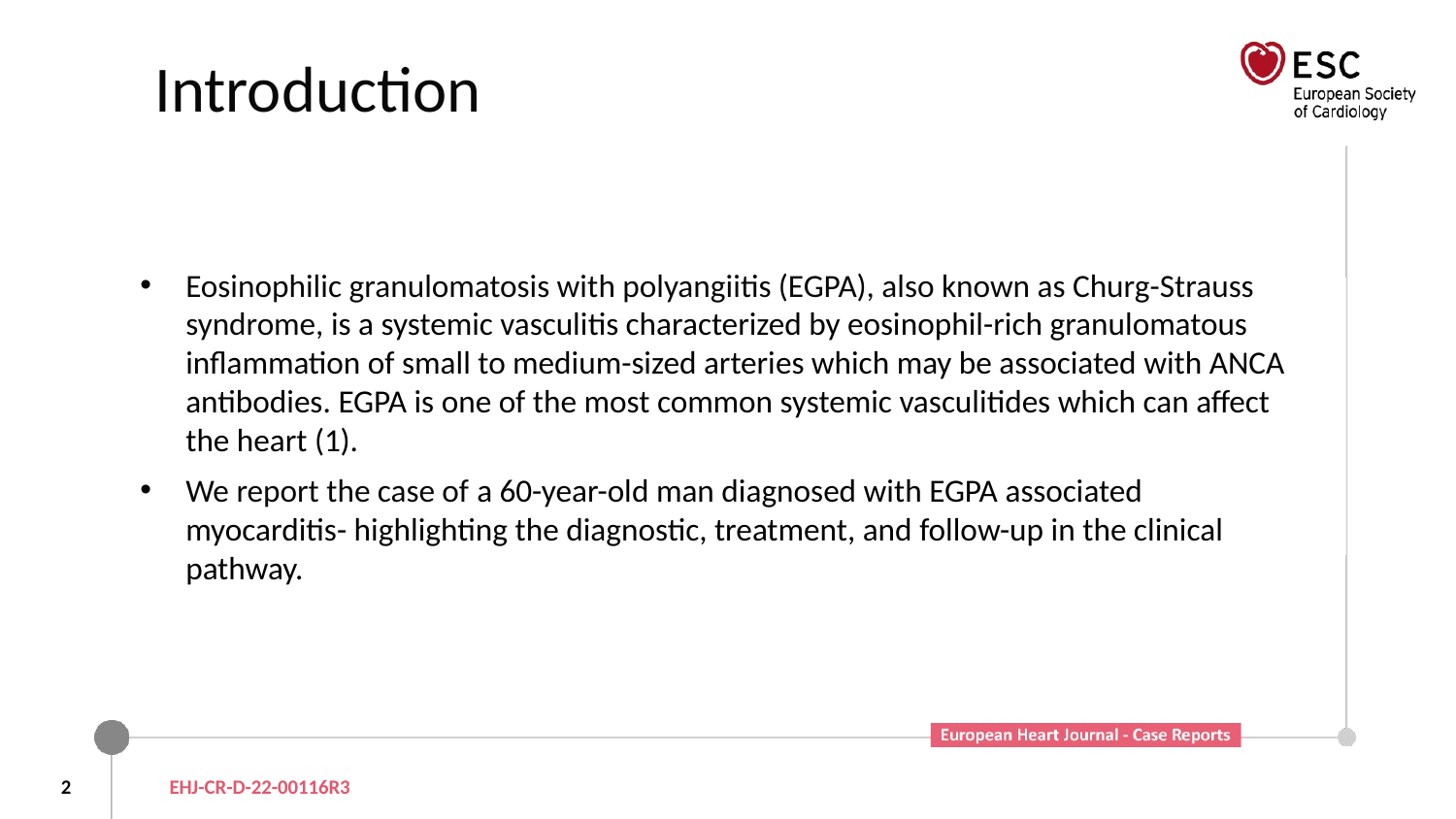

# Introduction
Eosinophilic granulomatosis with polyangiitis (EGPA), also known as Churg-Strauss syndrome, is a systemic vasculitis characterized by eosinophil-rich granulomatous inflammation of small to medium-sized arteries which may be associated with ANCA antibodies. EGPA is one of the most common systemic vasculitides which can affect the heart (1).
We report the case of a 60-year-old man diagnosed with EGPA associated myocarditis- highlighting the diagnostic, treatment, and follow-up in the clinical pathway.
2
EHJ-CR-D-22-00116R3

## Slide 3
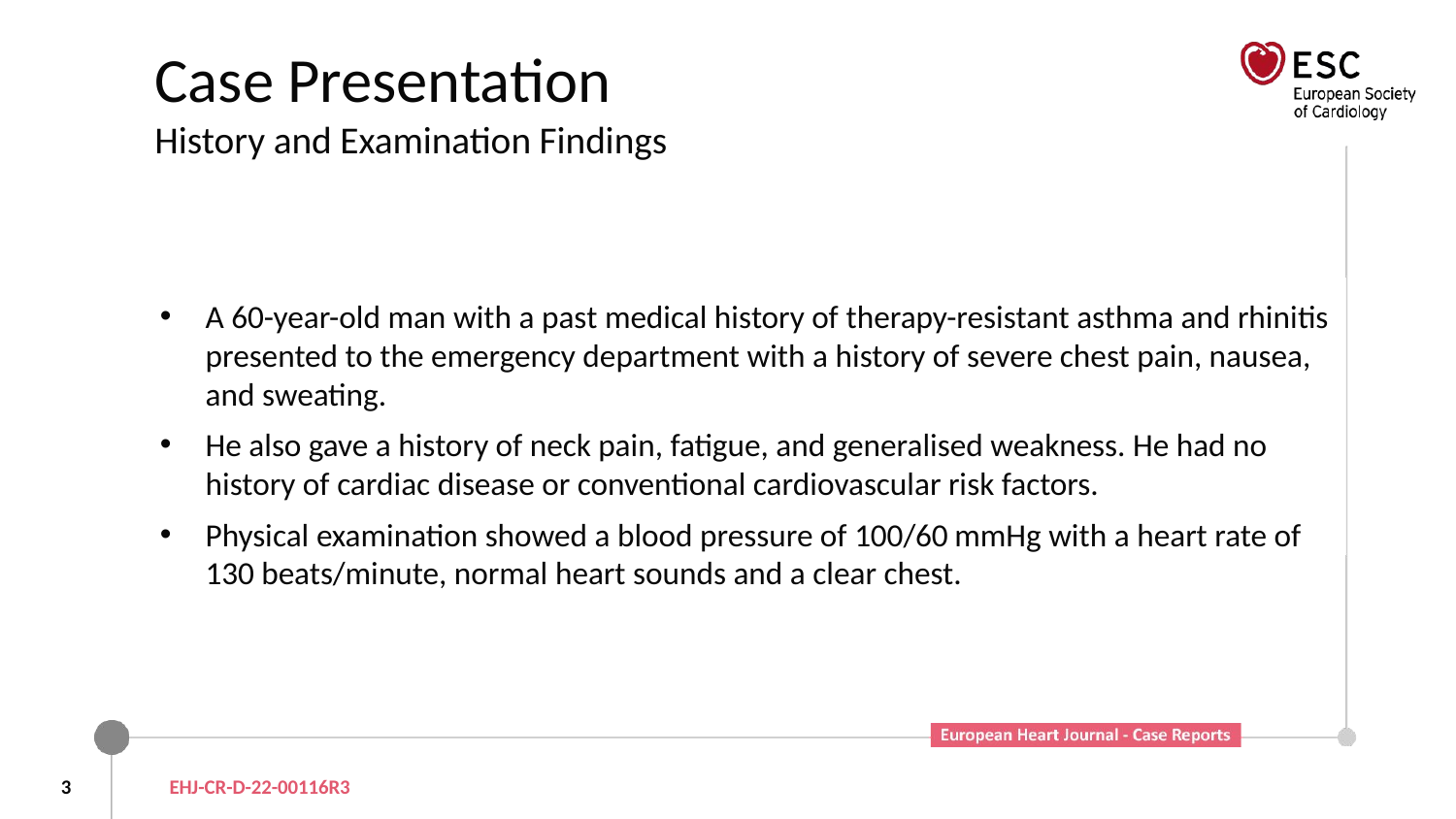

# Case Presentation History and Examination Findings
A 60-year-old man with a past medical history of therapy-resistant asthma and rhinitis presented to the emergency department with a history of severe chest pain, nausea, and sweating.
He also gave a history of neck pain, fatigue, and generalised weakness. He had no history of cardiac disease or conventional cardiovascular risk factors.
Physical examination showed a blood pressure of 100/60 mmHg with a heart rate of 130 beats/minute, normal heart sounds and a clear chest.
3
EHJ-CR-D-22-00116R3

## Slide 4
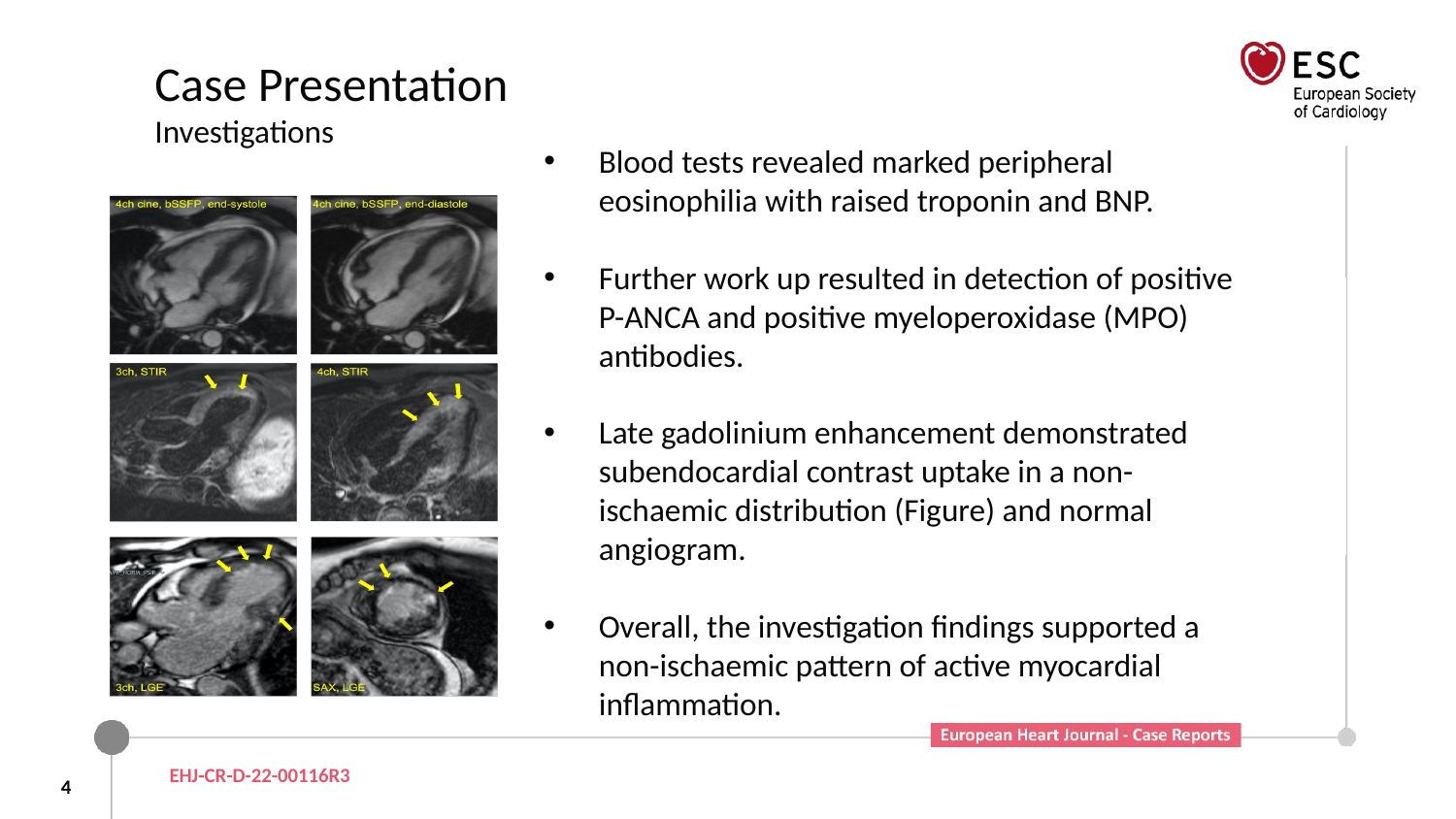

# Case Presentation Investigations
Blood tests revealed marked peripheral eosinophilia with raised troponin and BNP.
Further work up resulted in detection of positive P-ANCA and positive myeloperoxidase (MPO) antibodies.
Late gadolinium enhancement demonstrated subendocardial contrast uptake in a non-ischaemic distribution (Figure) and normal angiogram.
Overall, the investigation findings supported a non-ischaemic pattern of active myocardial inflammation.
4
EHJ-CR-D-22-00116R3

## Slide 5
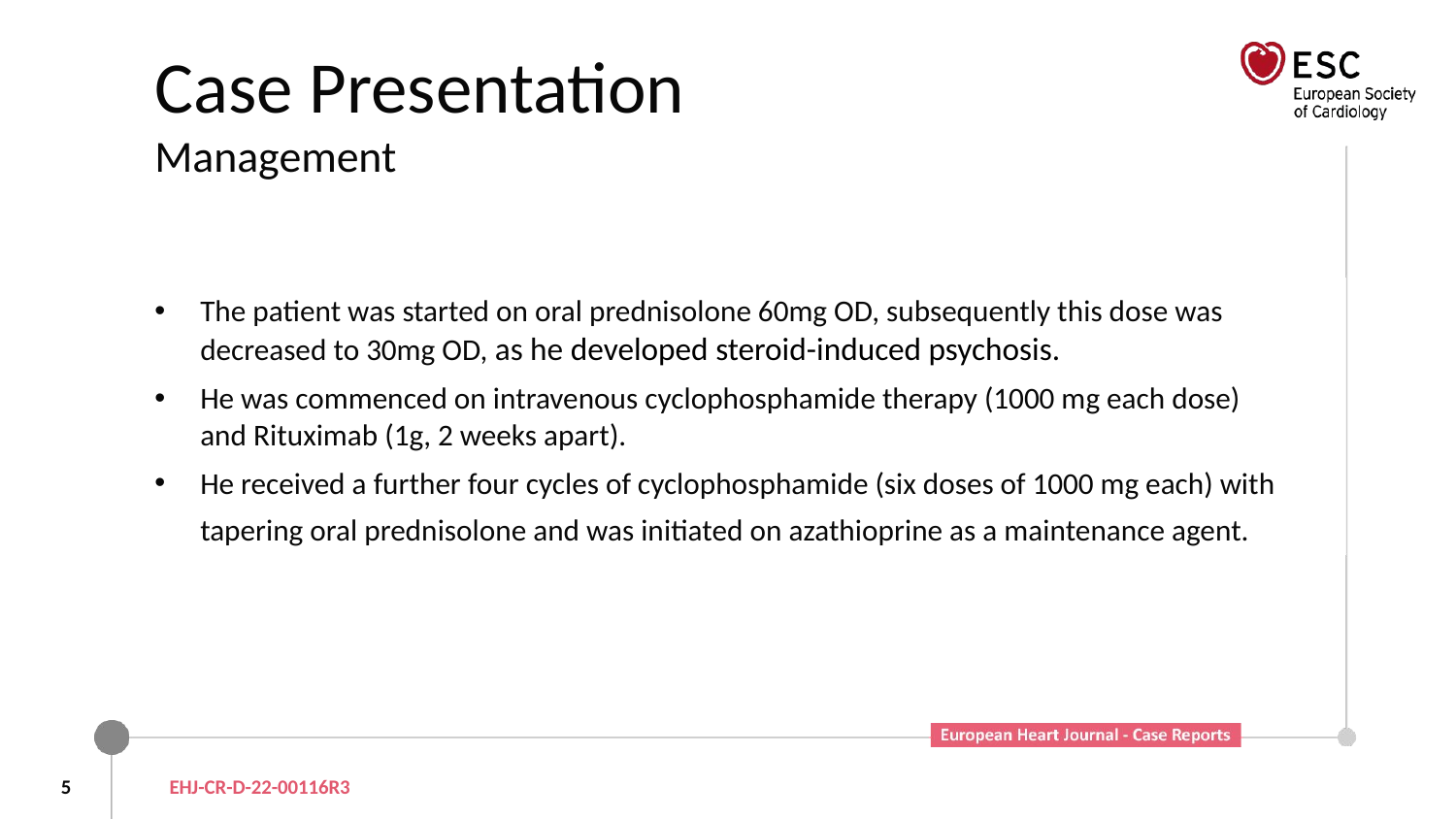

# Case Presentation Management
The patient was started on oral prednisolone 60mg OD, subsequently this dose was decreased to 30mg OD, as he developed steroid-induced psychosis.
He was commenced on intravenous cyclophosphamide therapy (1000 mg each dose) and Rituximab (1g, 2 weeks apart).
He received a further four cycles of cyclophosphamide (six doses of 1000 mg each) with tapering oral prednisolone and was initiated on azathioprine as a maintenance agent.
5
EHJ-CR-D-22-00116R3

## Slide 6
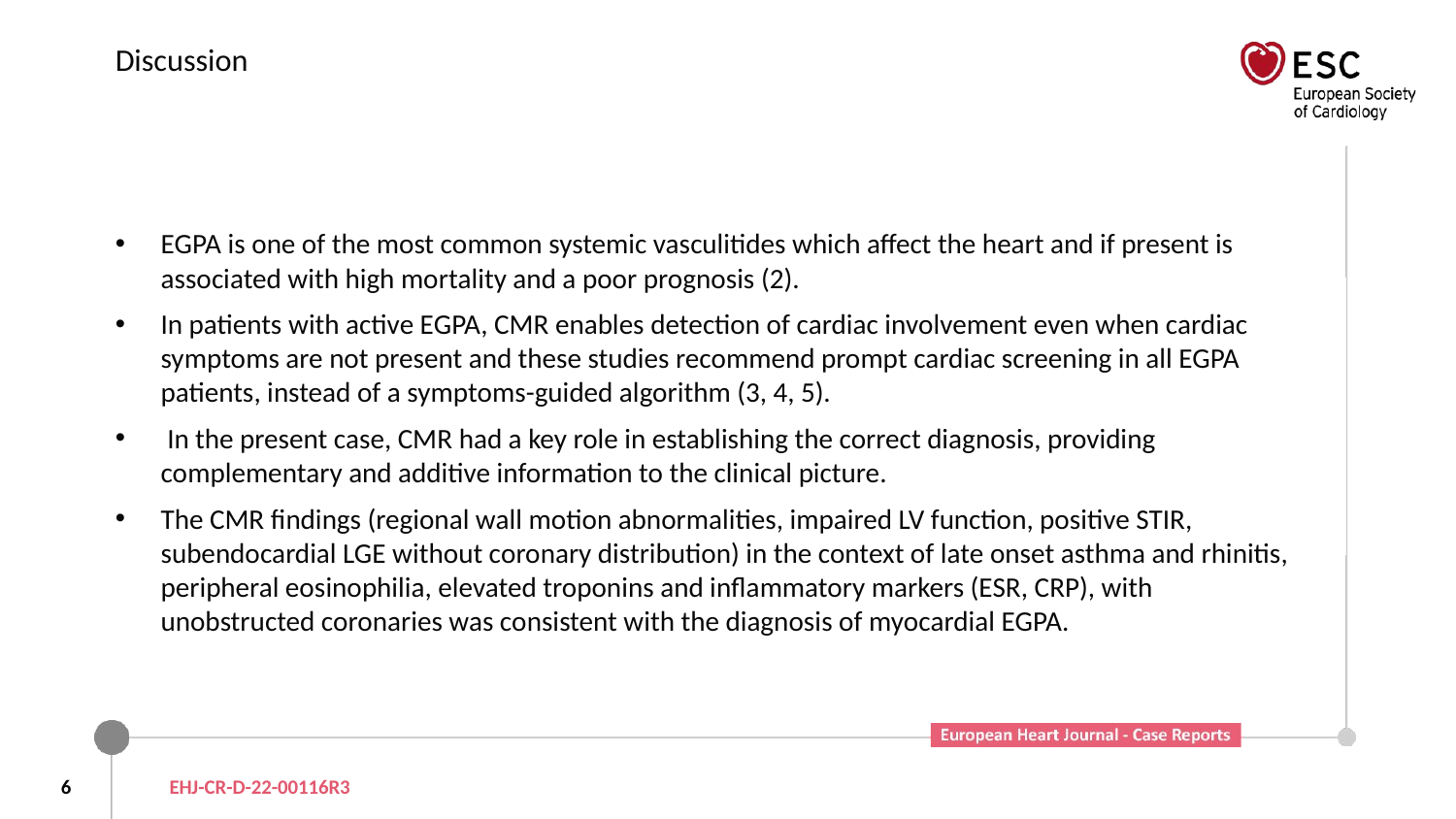

# Discussion
EGPA is one of the most common systemic vasculitides which affect the heart and if present is associated with high mortality and a poor prognosis (2).
In patients with active EGPA, CMR enables detection of cardiac involvement even when cardiac symptoms are not present and these studies recommend prompt cardiac screening in all EGPA patients, instead of a symptoms-guided algorithm (3, 4, 5).
 In the present case, CMR had a key role in establishing the correct diagnosis, providing complementary and additive information to the clinical picture.
The CMR findings (regional wall motion abnormalities, impaired LV function, positive STIR, subendocardial LGE without coronary distribution) in the context of late onset asthma and rhinitis, peripheral eosinophilia, elevated troponins and inflammatory markers (ESR, CRP), with unobstructed coronaries was consistent with the diagnosis of myocardial EGPA.
6
EHJ-CR-D-22-00116R3

## Slide 7
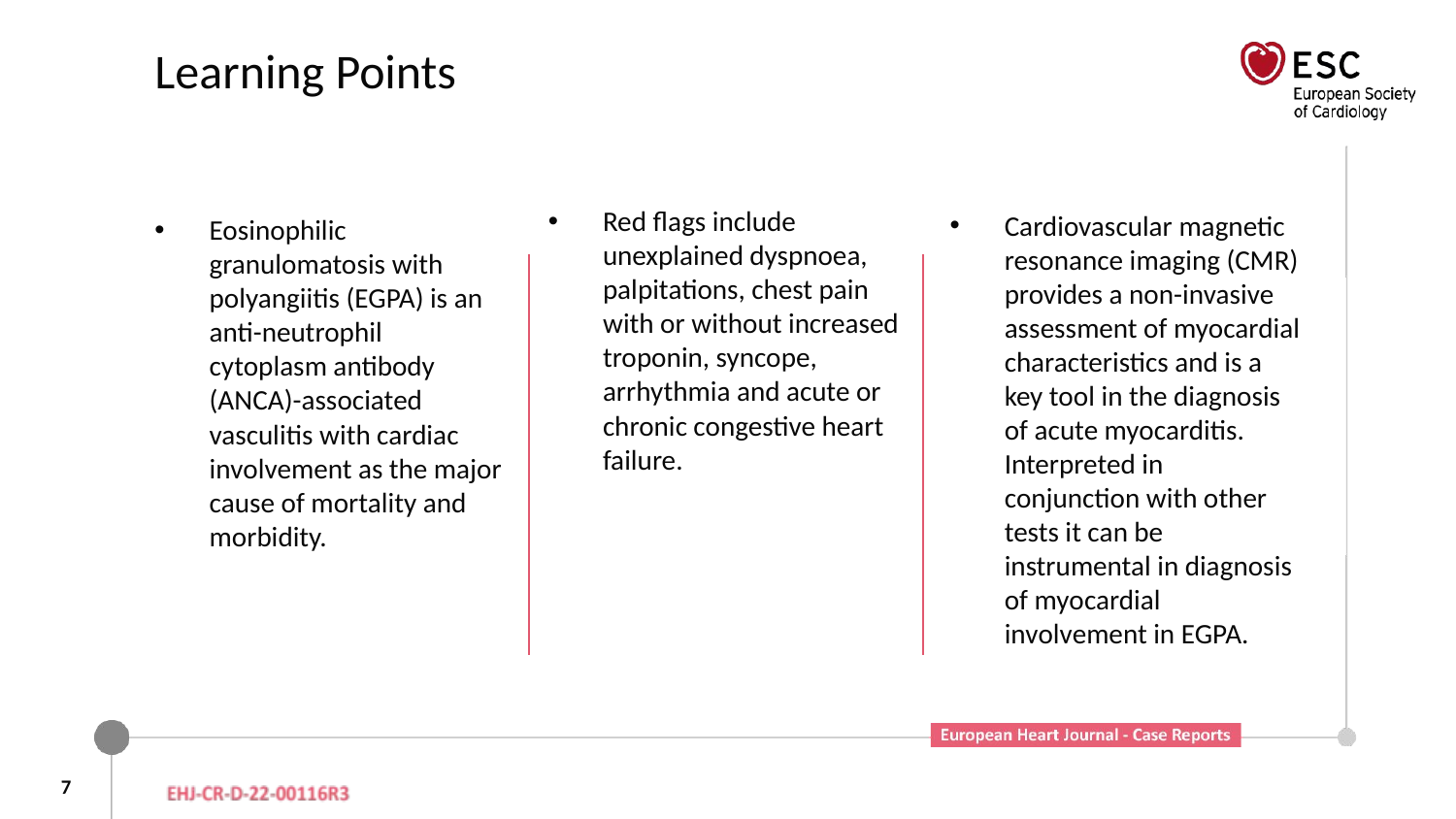

# Learning Points
Red flags include unexplained dyspnoea, palpitations, chest pain with or without increased troponin, syncope, arrhythmia and acute or chronic congestive heart failure.
Cardiovascular magnetic resonance imaging (CMR) provides a non-invasive assessment of myocardial characteristics and is a key tool in the diagnosis of acute myocarditis. Interpreted in conjunction with other tests it can be instrumental in diagnosis of myocardial involvement in EGPA.
Eosinophilic granulomatosis with polyangiitis (EGPA) is an anti-neutrophil cytoplasm antibody (ANCA)-associated vasculitis with cardiac involvement as the major cause of mortality and morbidity.
7

## Slide 8
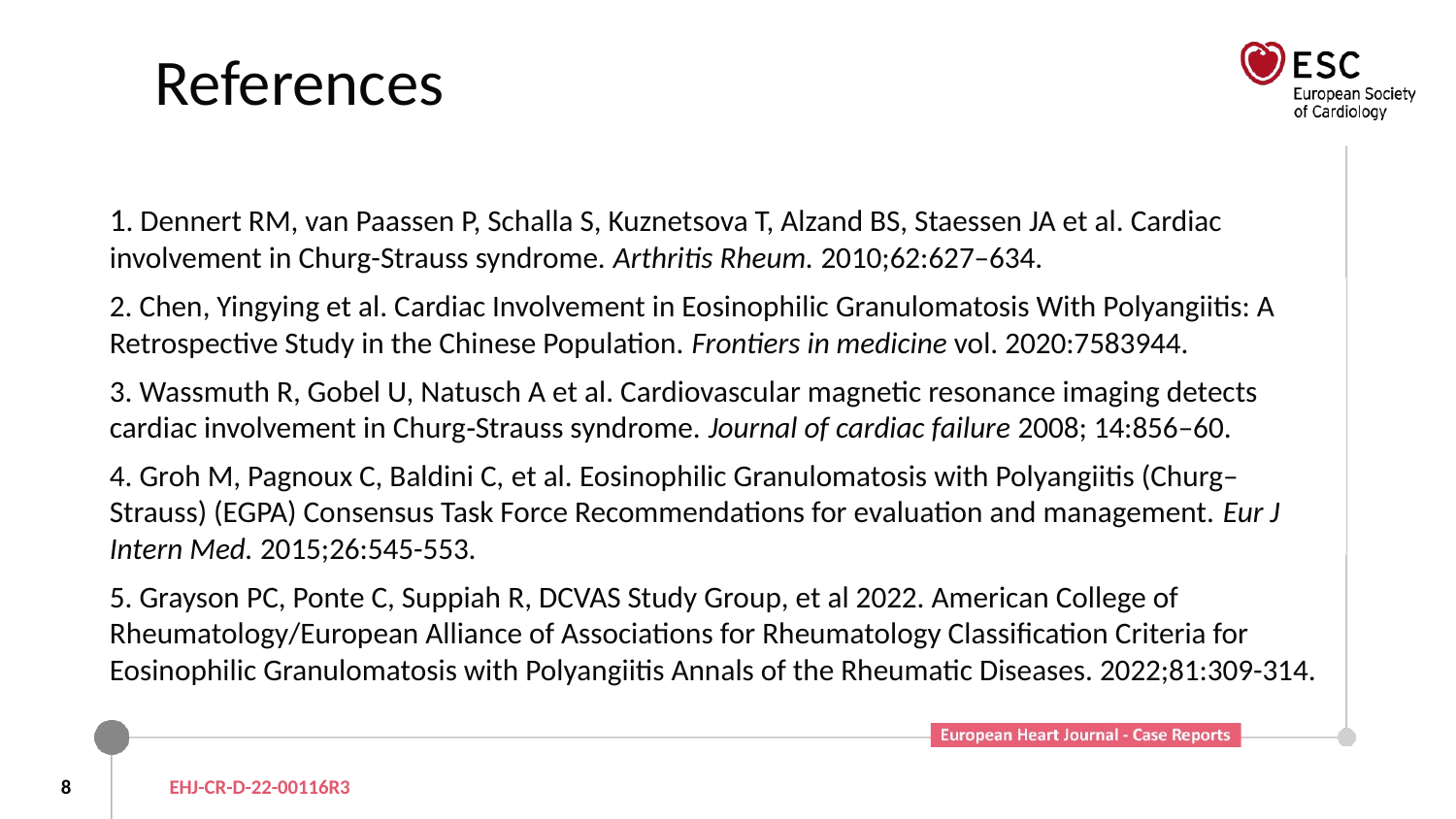

# References
1. Dennert RM, van Paassen P, Schalla S, Kuznetsova T, Alzand BS, Staessen JA et al. Cardiac involvement in Churg-Strauss syndrome. Arthritis Rheum. 2010;62:627–634.
2. Chen, Yingying et al. Cardiac Involvement in Eosinophilic Granulomatosis With Polyangiitis: A Retrospective Study in the Chinese Population. Frontiers in medicine vol. 2020:7583944.
3. Wassmuth R, Gobel U, Natusch A et al. Cardiovascular magnetic resonance imaging detects cardiac involvement in Churg‐Strauss syndrome. Journal of cardiac failure 2008; 14:856–60.
4. Groh M, Pagnoux C, Baldini C, et al. Eosinophilic Granulomatosis with Polyangiitis (Churg–Strauss) (EGPA) Consensus Task Force Recommendations for evaluation and management. Eur J Intern Med. 2015;26:545-553.
5. Grayson PC, Ponte C, Suppiah R, DCVAS Study Group, et al 2022. American College of Rheumatology/European Alliance of Associations for Rheumatology Classification Criteria for Eosinophilic Granulomatosis with Polyangiitis Annals of the Rheumatic Diseases. 2022;81:309-314.
8
EHJ-CR-D-22-00116R3
